# Nomenclatural benchmarking: the roles of digital typification and telemicroscopy

**DOI:** 10.3897/zookeys.209.3486

**Published:** 2012-07-20

**Authors:** Quentin Wheeler, Thierry Bourgoin, Jonathan Coddington, Timothy Gostony, Andrew Hamilton, Roy Larimer, Andrew Polaszek, Michael Schauff, M. Alma Solis

**Affiliations:** 1 International Institute for Species Exploration, Arizona State University, Tempe, AZ 85287 USA; 2 Laboratoire d’Entomologie, Museum National d’Histoire Naturelle, Rue Buffon, Paris, France; 3 Department of Life Sciences, The Natural History Museum, London SW7 5BD, U.K.; 4 United States Department of Agriculture, Systematic Entomology Laboratory, Beltsville, MD 20705 USA; 5 Visionary Digital, Palmyra, VA 22963 USA 6 National Museum of Natural History, Smithsonian Institution, Washington, DC 20530 USA; 6 National Museum of Natural History, Smithsonian Institution, Washington, DC 20530 USA

**Keywords:** Types, typification, digital imaging, biodiversity informatics, taxonomy, nomenclature, natural history museums

## Abstract

Nomenclatural benchmarking is the periodic realignment of species names with species theories and is necessary for the accurate and uniform use of Linnaean binominals in the face of changing species limits. Gaining access to types, often for little more than a cursory examination by an expert, is a major bottleneck in the advance and availability of biodiversity informatics. For the nearly two million described species it has been estimated that five to six million name-bearing type specimens exist, including those for synonymized binominals. Recognizing that examination of types in person will remain necessary in special cases, we propose a four-part strategy for opening access to types that relies heavily on digitization and that would eliminate much of the bottleneck: (1) modify codes of nomenclature to create registries of nomenclatural acts, such as the proposed ZooBank, that include a requirement for digital representations (e-types) for all newly described species to avoid adding to backlog; (2) an “r” strategy that would engineer and deploy a network of automated instruments capable of rapidly creating 3-D images of type specimens not requiring participation of taxon experts; (3) a “K” strategy using remotely operable microscopes to engage taxon experts in targeting and annotating informative characters of types to supplement and extend information content of rapidly acquired e-types, a process that can be done on an as-needed basis as in the normal course of revisionary taxonomy; and (4) creation of a global e-type archive associated with the commissions on nomenclature and species registries providing one-stop-shopping for e-types. We describe a first generation implementation of the “K” strategy that adapts current technology to create a network of Remotely Operable Benchmarkers Of Types (ROBOT) specifically engineered to handle the largest backlog of types, pinned insect specimens. The three initial instruments will be in the Smithsonian Institution(Washington, DC), Natural History Museum (London), and Museum National d’Histoire Naturelle (Paris), networking the three largest insect collections in the world with entomologists worldwide. These three instruments make possible remote examination, manipulation, and photography of types for more than 600,000 species. This is a cybertaxonomy demonstration project that we anticipate will lead to similar instruments for a wide range of museum specimens and objects as well as revolutionary changes in collaborative taxonomy and formal and public taxonomic education.

## Introduction

Our ability to explore, sustain, and utilize biodiversity depends on accurate species identifications, predictive phylogenetic classifications, and reliable scientific names. Biodiversity informatics relies on scientific names and the field continues to expand uses of binominals in information management and analysis (Patterson et al. 2006, 2010).

Species-level binominals are objectively applied due to the practice of typification in which a single specimen is designated to function as a representative or standard for the name (Blackwelder 1967, ICZN 1999, McNeill et al. 2006) Nomenclatural benchmarking is the periodic alignment of species names with changing theories of the limits of species and involves the reexamination of type specimens. Although the Code aims to promote stability in nomenclature Eugene Gaffney (1979) observed that taxonomic stability *is* ignorance. New data, specimens, and analyses inevitably change and improve our understanding of species. These changes variously require coining new names, redefining concepts attached to existing names, or resurrecting names from synonymy. Unless binominals keep pace with the growth of knowledge and changing concepts of species, their information content and reliability as tools of communication and data management decline over time.

The process of nomenclatural benchmarking is the examination of type specimens of all available species-group names (i.e., all species-group names meeting the requirements of the prevailing Code) to ascertain which currently accepted taxonomic species the specimen bearing the name falls within. Whichever species the type specimen falls within, there follows the name attached to it. Difficulties in accessing types to inform nomenclatural decisions is slowing progress in taxonomy and threatening the integrity of biodiversity databases. Digital representations of types or e-types are clearly a major part of the solution. Where detailed images of types exist many nomenclatural decisions can be made rapidly and efficiently. Botanists have led the way in the systematic digitization of types with impressively effective results from projects of individual herbaria to coordinated community projects (e.g., Global Plants Initiative, see www.botanischestaatssammlung.de/projects/GPI.html ). Zoologists are making progress, including specialized imaging techniques for unique specimen challenges (e.g., Berquist et al. 2012), but have major challenges ahead.

Here we address four issues that we regard as major challenges for nomenclatural benchmarking. First, there is the matter of a massive backlog. It has been estimated that the nearly two million currently recognized species (Chapman 2009) are accompanied, including names in synonymy, by perhaps five to six million name-bearing types. There is no tally of the number of type specimens that have been digitized to date, but it is at most a fraction of the backlog. Second, there is the issue of adding to the backlog through the description of new species. There is no formal requirement or expectation that types of the 18,000 or so species described each year be digitized. Third, there is a need for access to type specimens by experts in cases where existing digital images (e-types) fail to reveal characters in sufficient detail for definitive decisions regarding status. And, finally, there is a global need for a portal for access to all e-types.

We propose a strategy for addressing these challenges, including (I) modifications of the Codes to assure no further accumulation of backlogs of non-digitized types, (II) an “r” strategy that relies on automation to rapidly create reasonably informative e-types without the need for expert involvement; (III) a “K” strategy that engages experts to expand and refine such first approximation e-types; and (IV) the creation of a global archive of e-types. In addition, we describe a first generation “K” strategy instrument accessible via the Internet as part of an international network of remotely operable digital microscopes that make insect types accessible to taxon experts and that we anticipate will be launched in December, 2012.

### I: Digitize types for new species at time of description

We could avoid adding to an already massive backlog of un-digitized types by adopting a few simple practices. First, we believe that the Codes should be modified to mandate registration of all nomenclatural acts, including descriptions of new species (Polaszek et al. 2005). As a further requisite, e-types should be a mandatory part of the registration of new species. While the minimum requirement would be one or more images, authors should be urged to include both a habitus representation of the type, preferably from multiple angles, as well as additional annotated detailed images of diagnostic anatomical details. Successful implementation will require standards for images as well as for data and metadata capture and dissemination, but such standards are already in wide use in biological informatics and should pose no serious difficulty.

Major museums that accession large numbers of types each year should establish e-typification centers to meet their in-house needs and to serve as a regional digital typification center. E-types could be created at a nominal fee for taxonomists working outside such institutions or offered at no charge for authors willing to permanently deposit the type with the museum.

### II: Rapid (“r” Strategy) e-typification

To deal with a backlog of millions of type specimens we propose the development and engineering of automated e-typification instruments capable of rapidly capturing as much visual information from the specimen as possible without the need for expert intervention. It is easy to imagine such automated instruments that rotate the specimen, orbit a digital camera, or employ a battery of digital cameras to rapidly create rotatable and scalable 2D and 3D images of types. This would capture most, but not all, characters and provide a reasonably good first approximation of an e-type. Automation will result in low personnel costs. Deployed in numbers, such instruments could quickly eliminate the backlog. Following this initial digitization of the backlog these instruments could be permanently installed at the e-typification centers discussed above.

### III: Comprehensive (“K” Strategy) e-typfication

One reason that the “r” strategy is rapid is that it imposes a one-size-fits-all approach to creating reasonably good 3D composites of type specimens. While resulting e-types will enable many nomenclatural decisions, in other cases the images will be found wanting in detail, illumination, angle, or some other respect. In certain cases, such as where a dissection is necessary to reveal a character, a physical visit to the museum or shipment of a specimen is unavoidable. In other cases it may be that simply connecting an expert with a type specimen via telemicroscopy is enough. This “K” strategy takes advantage of expert knowledge to supplement existing images with those that target diagnostic characters. This is a symbiotic relationship, with the expert gaining precious access to a type and the museum profiting from expert knowledge, because the images captured from telemicroscopy will become part of the composite e-type.

Benefits of telemicroscopy are obvious. They can save a great deal of time and money compared to visits by experts to museums, they can virtually repatriate types to scientists in countries of origin allowing a level of interaction not possible with archived images, they can dramatically decrease wear and tear on specimens, and they further democratize taxonomy by leveling the field for amateurs and scientists at small institutions who will have equal access to types.

### IV: A global e-type archive

A comprehensive, distributed, open-access global e-type archive is urgently needed. In fact, next to completing a catalog with the status of all available species names, such an archive ranks among the greatest needs for advancing biodiversity exploration and informatics. A global e-type archive would provide one-stop access to images of the type specimens for any species and would be complementary to, and possibly accessible through, portals such as ZooBank, the Encyclopedia of Life, and the Biodiversity Heritage Library. It could also be easily hyperlinked in electronic taxonomic journals and monographs.

**Figure 1.**
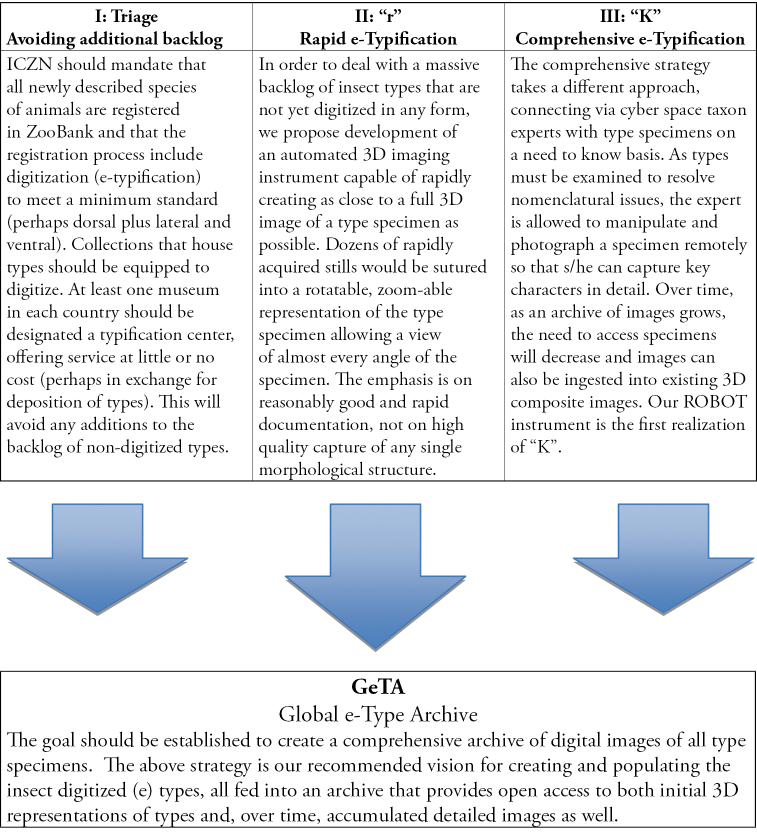
Three-part strategy to (a) avoid further growth of backlog by digitizing all new species, (b) rapidly create 3D e-types for all existing species, and (c) open access to types for experts to facilitate their nomenclatural decision-making while simultaneously expanding and enhancing comprehensiveness of digital images of informative characters of type specimens. All images should be available through an open access public “Global e-Type Archive,” whether managed by ZooBank or a community-level organization.

## Implementing “K” strategy for insect type specimens

### ROBOT(E)

The idea of sharing specialized research instruments through Web access is not new (Hadida-Hassan et al. 1999) and, in our case, can be expanded to include specialized research resources such as specimens in collections. Histologists and pathologists have used telemicroscopy for decades and pioneered many innovative applications including robotic controls, archival images, multiple simultaneous viewing, interdisciplinary telecommunication, team consultation, and expert teleconsultation (e.g., Bellina and Missoni 2009, Leong and McGee 2001, Mea et al. 1999, Kayser 2002, Pantanowitz 2010) with application by extension to taxonomy.

Networking three leading insect collections in Washington, DC (Smithsonian Institution, National Museum of Natural History, Department of Entomology), London (Natural History Museum, Department of Entomology), and Paris (Museum National d’Histoire Naturelle, Laboratoire d’Entomologie) we set out to demonstrate that telemicroscopy could be used to implement our “K” strategy. With just these three nodes in a network of remotely operable microscopes in a network scheduled to go "live" in December, 2012 we will open potential access to a large fraction of insect type specimens. These three collections, the largest on earth, contain more than 600,000 insect type specimens and more than 100,000,000 specimens possibly representing as many as 80% or more of known insect species.

Our selection of insects for a demonstration project was a relatively easy one for several reasons. First, insects account for more than one million described (Foottit and Adler 2009, Zhang 2011) species and an estimated two to three million type specimens. Second, many types are preserved as dry, pin-mounted specimens, making the engineering challenge of handling them manageable. Third, many types fall within a reasonable size range, again easing the challenge of handling most of them with a single device. Finally, many insects are of great agricultural, medical, and ecological interest and their taxonomy is undergoing rapid change requiring frequent access to types.

**Figure 2.**
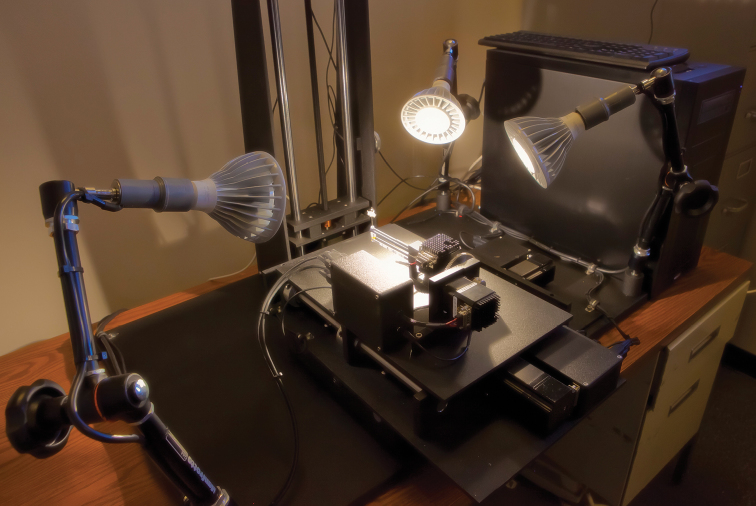
Two ROBOT(E) remotely operable digital imaging systems designed to allow taxonomists to examine, manipulate, and digitally photograph type specimens through a Web connection. Three such instruments are being deployed to major insect collections in Washington, London, and Paris. A prototype instrument remains with the IISE for testing and development purposes. PHOTO: Courtesy of Erik Holsinger, Arizona State University.

We have named our system ROBOT (Remotely Operable Benchmarker Of Types), with the first iteration (E) specially designed to handle pinned entomological types. Our goal was to make the system as simple and reliable as possible and to minimize costs by using as much off-the-shelf technology as feasible. The heart of ROBOT(E) is a digital Canon 7D camera that gave us several critically important capabilities beyond capturing images including auto-focus and through-the-sensor high resolution viewing. For the *z* axis we used the Visionary Digital BK P-51 CamLift that has a very precise linear actuator that can be moved in increments as small as 6.0 microns. The *x* and *y* axes use precise micro-step motors to move plates that were custom manufactured by a machine shop. Heavy studio-style lamp holders were modified to secure daylight temperature (ca. 5000 K) LED lamps that would operate on 120 or 240 v current. For the specimen holder, we designed an arm linked to two additional micro-step motors so that the specimen may be spun 360 degrees and “rolled” 180 degrees to reveal the ventral surfaces of specimens. The pin is secured by a tight bundle of fine acrylic cable into which it is inserted.

We designed and wrote the ROBOT(E) software to be simple and intuitive. Several “windows” may be seen or hidden and resized or positioned to meet user preferences. Simple mouse, arrow key, and button choices operate the system’s five motors. Autofocus may be alternated with fine manual focusing. Autofocus is disabled when the specimen is rolled, and an algorithm keeps the specimen in approximate focus. Images are stored in a temporary folder from which they may be downloaded to any target folder. In addition, users may create bookmarks that remember *x*, *y*, and *z* coordinates so that specific views may quickly be recovered.

This first generation of ROBOT is intended to prove the usefulness of telemicroscopy in the study of types and has limitations. Future generations could easily be modified to handle a range of museum specimens or objects with little modification. Once the systems are fully tested in museum settings, we plan to add a number of additional features, including an automated image stacking montage function and improved control over illumination. Options will likely include a choice of spot or diffuse light. By combining ROBOT with an advanced video communication software package, colleagues can examine a type or rare specimen simultaneously, a specimen intercepted at a port of entry could be identified in consultation with an expert, or an expert could use the specimen for advanced teaching. We hope that this project serves to encourage additional uses for remote microscopy and paves the way to open access to types.

**Figure 3.**
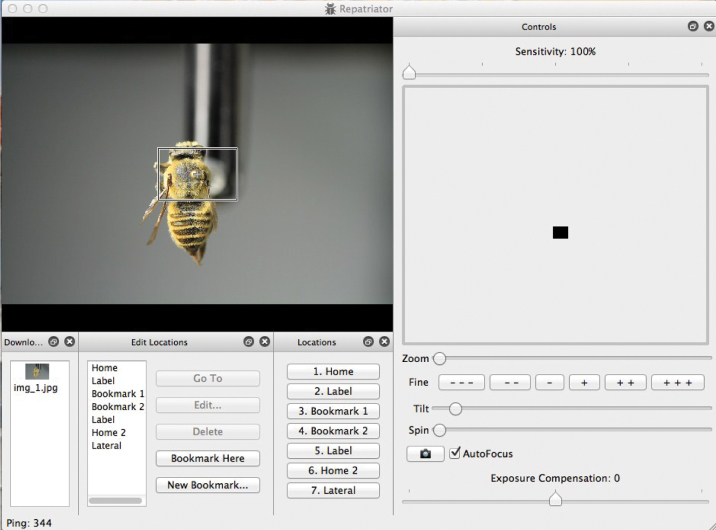
Screen capture of ROBOT(E) system in use. User is able to orient specimen on multiple axes by actuating micro-step motors that position on *x*, *y*, and *z* scales as well as spinning around axis of pin or tilting specimen to examine lateral or ventral perspectives.

## Conclusions

Our implementation of a network of remotely operable digital microscopes serves as a demonstration that high value specimens can be accessed, examined and imaged from virtually anywhere. It is merely one step in the modernization of museum specimen access. This is not a general solution to type accessibility or a substitute for creating e-types. We propose a broader strategy of which this direct connection of expert and type is merely one component. Our other recommendations include a global archive of type images, e-typification at time of original description and registration, and engineering automated instruments to rapidly create 3D images of all types. We also foresee modifications to improve our telemicroscopes in terms of their functionality, ability to handle a wide range of specimens and objects, and coupling with automated systems that alleviate much of the need for human involvement in specimen access.

## References

[B16] Agosti A Alonso-Zarazaga M Beccaloni G de Place Bjørn P Bouchet P Brothers DJ the Earl of Cranbrook Evenhuis N Godfray HCJ Johnson NF Krell F-T Lipscomb D Lyal CHC Mace GM Mawatari S Miller S Minelli A Morris S Ng PKL Patterson DJ Pyle RL Robinson N Rogo L Thompson FC van Tol J Wheeler QD Wilson EQ (2005) A universal register for animal names. Nature 437: 477. doi: 10.1038/437477a 16177765

[B1] Bellina L Missoni E (2009) Mobile cell-phones (M-phones) in telemicroscopy: increasing connectivity of isolated laboratories. Diagnostic Pathology 4: 19. doi: 10.1186/1746-1596-4-19 10.1186/1746-1596-4-19 PMC2706795 19545373

[B2] Berquist RM Gledhill KM Peterson MW Doan AH Baxter GT Yopak KE Kang N Walker HJ Hastings PA Frank LR (2012) The digital fish library: Using MRI to digitize, database, and document the morphological diversity of fish. PLoS ONE 7: e34499. doi: 10.1371/journal.pone.0034499 PMC3321017 22493695

[B3] Blackwelder RE (1967) Taxonomy: A Text and Reference Book. Wiley, New York. 714 pp.

[B4] Chapman AD (2009) Numbers of Living Species in Australia and the World. Australian Biological Resources Study, Canberra. 80 pp.

[B5] Foottit RG Adler PH (Eds) (2009) Insect Biodiversity: Science and Society. Wiley-Blackwell, Chichester, 632 pp.

[B6] Gaffney ES (1979) An introduction to the logic of phylogeny reconstruction. In: Cracraft J Eldredge N (Eds). Phylogenetic Analysis and Paleontology. Columbia University Press, New York: 79-111.

[B7] Hadida-Hassan M Young SJ Peltier ST Wong M Lamont S Ellisman MH (1999) Web-based telemicroscopy. Journal of Structural Biology 125: 235-245. doi: 10.1006/jsbi.1999.4095 10222280

[B8] ICZN (1999) International Code of Zoological Nomenclature. 4^th^ ed. International Trust for Zoological Nomenclature, London, 306 pp.

[B9] Kayser K (2002) Interdisciplinary telecommunication and expert teleconsultation in diagnostic pathology: present status and future prospects. Journal of Telemedicine and Telecare 8: 325-330. doi: 10.1258/135763302320939202 12537919

[B10] Leong FJW-M McGee JO’D (2001) Automated complete slide digitization: a medium for simultaneous viewing by multiple pathologists. Journal of Pathology 195: 508-514. doi: 10.1002/path.972 11745684

[B11] McNeill J Barrie FR Burdet HM Demoulin V Hawksworth DL Marhold K Nicolson DH Prado J Silva PC Skog JE Wiersema JH Turland NJ (Eds) (2006) International Code of Botanical Nomenclature (Vienna Code). Regnum Vegetabile 146. A. R. G. Gantner Verlag KG., Ruggell, 568 pp.

[B12] Mea VD Cataldi P Pertoldi B Beltrami CA (1999) Dynamic robotic telepathology: a preliminary evaluation on frozen sections, histology and cytology. Journal of Telemedicine and Telecare 5: 55-56. doi: 10.1258/1357633991932559 10534842

[B13] Pantanowitz L (2010) Digital images and the future of digital pathology. Journal of Pathological Informatics 1: 15. doi: 10.4103/2153-3539.68332 PMC2941968 20922032

[B14] Patterson DJ Remsen D Marino WA Norton C (2006) Taxonomic indexing — extending the role of taxonomy. Systematic Biology 55: 367-373. doi: 10.1080/10635150500541680 16861205

[B15] Patterson DJ Cooper J Kirk P Pyle R Remsen DP (2010) Names are key to the big new biology. Trends in Ecology and Evolution 25: 686-691. doi: 10.1016/j.tree.2010.09.004 20961649

[B17] Zhang Z-Q (2011) Phylum Arthropoda von Siebold, 1848 In: Zhang Z-Q (Ed). Animal biodiversity: An outline of higher-level classification and survey of taxonomic richness. Zootaxa 3148: 99-103. 10.11646/zootaxa.3703.1.1 26146682

